# Improving the uptake of preconception care and periconceptional folate supplementation: what do women think?

**DOI:** 10.1186/1471-2458-10-786

**Published:** 2010-12-23

**Authors:** Danielle Mazza, Anna Chapman

**Affiliations:** 1 Department of General Practice, Monash University, Building 1, 270 Ferntree Gully Road, Notting Hill, VIC, 3168, Australia

## Abstract

**Background:**

Despite strong evidence of the benefits of preconception interventions to improve pregnancy outcomes, the delivery and uptake of preconception care in general and periconceptional folate supplementation in particular remains low. The aim of this study was to determine women's views of the barriers and enablers to the uptake of preconception care and periconceptional folate supplementation.

**Methods:**

Focus groups were undertaken in 2007 with 17 women of reproductive age (18-45 years). To identify key issues and themes within the data, focus groups were analysed using an inductive process of thematic analysis.

**Results:**

Most women were unaware of the need to attend for preconception care and were surprised at the breadth of issues involved. Women also felt general practitioners (GPs) should be more proactive in promoting preconception care availability but acknowledged that they themselves had to be thinking about pregnancy or becoming pregnant to be receptive to it. Barriers to periconceptional folate supplementation included confusion about reasons for use, dose, duration, timing and efficacy of folate use. Enablers included the desire to do anything they could to ensure optimum pregnancy outcomes, and promotional material and letters of invitation from their GP to advise them of the availability and the need for preconception care.

**Conclusion:**

A number of important barriers and enablers exist for women regarding the delivery and uptake of preconception care and periconceptional folate supplementation. It is essential that these patient perspectives are addressed in both the implementation of evidence based clinical practice guidelines and in the systematic design of an intervention to improve preconception care delivery.

## Background

The repeated findings of low levels of knowledge and behaviour related to preconception care in women of reproductive age [1-4], particularly with regards to adherence to nutrition and lifestyle recommendations for planning a pregnancy [4] gives rise to questions about the efficacy of public health campaigns aimed at influencing these factors [5].

Women report that they prefer to obtain preconception advice from their primary care physician, yet only 39% of women recall doing so [6]. This figure suggests that doctors do not routinely address preconception care in practice or implement existing preconception care guidelines, and also indicates that women do not actively seek such care.

While research to date has provided insights into the factors associated with lower rates of folic acid supplement use, such as unintended pregnancy, age, socio-economic status and ethnic group [1,7,8], there has been little published regarding women's views or any exploration of barriers and enablers to the delivery and uptake of preconception care and periconceptional folate supplementation. By ignoring these views we may neglect aspects of care provision which are important from the perspective of consumers of health care [9].

Preconception care guidelines and recommendations have been developed in many countries [10-12]. In order to capitalise on the preventive 'window of opportunity' of pregnancy, further information is needed to understand how to best implement preconception care guidelines [5]. As part of a larger study to develop and evaluate an intervention to improve the delivery of preconception care, our aim was to determine women's views on the barriers and enablers to preconception care uptake and periconceptional folate supplementation.

## Methods

### Study participants

Three focus groups were undertaken between October and November 2007 with 17 women of reproductive age (18-45 years). The total number of focus groups was limited, as their primary aim was to inform the next phase of the study and to complement focus groups undertaken with GPs. Focus groups took place in a low and high socioeconomic area of metropolitan Melbourne and in South Gippsland, a rural area of Victoria, Australia. We used the Index of Relative Socioeconomic Disadvantage [13] to determine postcodes in the lowest and highest quartiles of the index and recruited women residing at these postcodes through advertisements in local newspapers. Participants in our rural focus group were obtained through a convenience sample at a local playgroup and Division of General Practice due to a lack of response to the newspaper advertisement. Each focus group consisted of 5-7 participants.

### Data Collection

The objectives and format of the focus groups were explained to participants before commencement, and their anonymity was assured. Participants gave written consent to participate. To maintain consistency, all focus groups were conducted by the same facilitator (DM) in a conversation-like manner and followed a schedule of guiding questions (Table 1). The study was approved by the Monash University Standing Committee on Ethics in Research Involving Humans.

**Table 1:** Schedule of guiding questions used in the focus groups

1.	What triggered your interest in this subject?
2.	To date, what has been your experience with preconception care?
3a.	What do you know about folate and pregnancy?
3b.	What are neural tube defects e.g. spina bifida?
3c.	Is dietary folate adequate in meeting women's needs for pregnancy?
3d.	How much folate (mg) should women take and when should they take it to prevent neural tube defects?
4.	What other health issues need to be addressed prior to pregnancy?
5.	Where do you go to find out information about folate and preconception health care issues?
6.	What issues prevent women from using folate supplements?
7.	What would assist women to use folate prior to conception?
8.	What is the role of a General Practitioner (GP) in preconception care?
9.	Are GPs conducting preconception care at the moment?
10.	How should GPs discuss preconception care issues with women?
11.	What issues may exist for women from culturally and linguistically diverse groups?
12.	What about women who are between pregnancies or who have previously had one or two children? Should anything different be offered to them?
13.	Is there anything important that hasn't already been raised?

### Data analysis

Data from the focus groups were audio-taped, transcribed verbatim and entered into NVIVO 7 software [14] to organise the data. Initially, transcripts were read and re-read by both authors in order to familiarise themselves with the data. To increase rigour, each transcript was independently coded line by line by both authors. An inductive process of thematic analysis, as described by Braun and Clarke [15], was employed to identify key issues and themes within the data. For areas where coding differed, agreement of interpretation was reached through meetings between both authors. The thematic results were then presented to the project advisory group (which included content and methodological experts) for discussion and further interpretation.

Quotes representing typical views expressed by the women were extracted from the transcripts and are presented in the results to illustrate the themes identified.

## Results

### Barriers

Four major barriers to the uptake of preconception care were found and one barrier to the uptake of periconceptional folate supplementation was consistently identified. With the exception of service provider issues, all barriers identified were consistent across groups.

### Degree of receptivity

There was a strong sense that women had to be receptive to the information in order to act upon it. This receptivity was dependent on their life stage and whether they were thinking about getting pregnant soon.

"You have to be in the zone." (Rural, 29 years)

Women who had already had a child felt they already knew about preconception care issues and didn't need to access preconception care.

"Because you think you know it. I don't have a child with spina bifida, so why should I take the folate? And I don't need to go the doctor because I've done it all before." (Low SES, 34 years)

### Conception is a normal event

Because conception is perceived as an event that most women go through, women felt that it was normal and that there was no need for medical attention or intervention prior to it occurring.

"Some women also think it's a natural thing; that we should just go ahead and get pregnant and all have healthy babies because that is the norm and is what's expected." (Low SES, 34 years)

Interestingly this contrasted with women's sense that it was important to present to a general practitioner (GP) as soon as they knew they were pregnant as they felt medical attention then became necessary.

"I thought the importance [of presenting to a GP] was more on when you were pregnant. When you think about going [to a GP], it's once you have fallen pregnant." (Rural, 29 years)

### Perceived risk and lack of awareness of the need for preconception care

Most women were unaware of the need for preconception care in general and said that their GPs did not offer preconception care or inform them of its availability. They were surprised at the breadth of issues that could comprise a preconception care consultation.

"It's a bit daunting when you get all this [preconception care information]. It's a lot to take in, but this is what I would like to have gotten from my GP. The thing is - I never had the concept of preconception care in my mind." (Rural, 26 years)

"Unless you actually go and pursue it [preconception care consultation] nothing will happen. (Low SES, 21 years)

### Service Provider Issues

Service provider issues were a major theme elicited in the focus groups and the theme most related to socioeconomic status and rurality. Women living in high socioeconomic areas described attending alternative health practitioners such as naturopaths and Chinese medical practitioners for preconception advice. This was particularly the case for women who had experienced trouble conceiving or who were currently accessing assisted reproductive technologies such as in-vitro fertilisation. Many women had been given specially prepared supplements by their naturopath but were unaware of the contents of these supplements. They nevertheless had implicit trust that they contained *'all that was needed' *to optimise conception and pregnancy outcomes.

"I don't have a GP; I have a naturopath, and have a really close relationship. She's been my guiding force behind all the information." (High SES, 33 years)

"If I was looking at information from a dietary perspective I would go to a naturopath. I would ask a GP but that's not their area of specialty and they're not so keen on supplements and vitamins." (High SES, 24 years)

"I am trying lots of things. I'm seeing a Chinese medicine specialist and he knows all the medications - more than my GP. I'm impressed about what he knows."(High SES, 40 years)

For a number of women the GP was not initially thought of as a major provider of preconception care. The reasons given for this were many. In rural areas there was difficulty accessing GPs, cost was a barrier for some women and women across all groups felt that as they were young and healthy, and in some cases yet to have children, they had not established an ongoing relationship with any particular GP and did not identify anyone as 'their GP'. Women also perceived the role of the GP to be one of 'acute care' and did not consider that GPs could or should deliver preventive care. Many felt that other patients who were unwell should have priority in a stretched system.

"You go to a GP because you're sick, not to discuss family planning." (High SES, 40 years)

"If I went in there to have a chat about planning pregnancy, would I be wasting their time?"(Rural, 26 years)

Women also felt that because there wasn't a procedure to carry out (in contrast to having a pap smear), they didn't feel they had to attend their GP to obtain preconception care advice.

"Because it's not active, with your pap smear and breast check you get something done, but for preconception care it's an appointment to go and talk, it's not actually a procedure." (Rural, 35 years)

Women's main source of information regarding preconception care was from friends and family and increasingly from the internet. Broadcast and printed media were not raised by participants.

"If I wanted information like that [preconception care] I would probably just go to an internet source...I wouldn't think of necessarily going to the GP if I can read it elsewhere." (Rural 26 years)

The most widely named internet sites being accessed for preconception related information were sites run by commercial for profit companies (media companies, companies producing nappies and those producing preconception supplements).

### Nature and symbolism of folate supplements

Most women expressed confusion about periconceptional folate supplementation: the dose, timing and benefits. There was particular confusion about the nature and efficacy of branded 'preconception' products and how these compared to supermarket brand products. They also voiced concern about the cost of vitamins. The purchase of folate supplements was for many women a clear marker of intent to conceive and they felt that keeping them on a shelf at home was an indication or sign to their partners that they were intending to conceive.

"That was one of the things; I just bought them [supplements] from the supermarket and they all had different milligrams. Do you have a multivitamin or just straight folate? I didn't know which one was better." (Rural, 29 years)

"I've always bought them [folate supplements] and had them ready to go but never really knew why." (Low SES, 36 years)

### Enablers

Two major thematic areas were identified in relation to enablers to the uptake of preconception care and periconceptional folate supplementation.

### High motivation to optimise pregnancy outcome

In relation to enablers of delivery and uptake of preconception care and periconceptional folate supplementation, there was universal agreement that women had a strong desire to achieve the best outcome possible for their baby and as such were very motivated to access information and engage in preventive care.

"I was really concerned; I wanted to do everything that I could for its [the baby's] welfare." (Rural, 32 years)

### Proactive promotion by GPs

While there was general agreement that public health campaigns and the media had a large role to play in raising awareness of folate supplementation and other preconception issues, there was strong support for GPs to be more proactive in making known the availability and need for preconception care.

"If they said to me if you think you might ever want to start planning a family these are some of the things we can discuss with you...I would file that away and when I'd made my decision I would come to talk to them about it." (Rural, 35 years)

Suggestions made were that during consultations for other matters GPs should discuss the availability and need for preconception care and suggest that women make another appointment to return. There were other suggestions made such as sending women of reproductive age a letter from the GP inviting attendance for preconception care, running preconception classes similar to 'antenatal classes', having preconception appointments available with a nurse, having posters in the waiting room to advertise preconception care and having information and patient brochures in waiting rooms.

## Discussion

This qualitative study provides new insights into the views of women of reproductive age regarding the barriers and enablers to the delivery and uptake preconception care and periconceptional folate supplementation, taking into account the significant variables of socioeconomic status and rurality. Although a relatively small sample of women was recruited to this study, the findings may give future direction to strategies for the implementation of preconception care guidelines in general practice.

Our findings, consistent with other studies [16] indicate a great willingness on the part of women to optimise their health in preparation for pregnancy; however several factors are acting as barriers. Major amongst these are the lack of GP 'push' for preconception care, with women reporting that GPs do not inform women of either the need for preconception care or its availability. Additionally, there is lack of patient 'pull' for these services because of a lack of familiarity with the concept of preconception care, the breadth of issues to be covered as part of preconception care or of its availability through general practice. Furthermore, whilst most women felt obligated to present to a GP once pregnant, a number of women stated that the concept of preconception care over-medicalised a natural human event. This dissonance of resisting the medicalisation of planning the occurrence of pregnancy whilst supporting it once pregnancy occurs may comprise a barrier that, if not more fully understood, will continue to impede the success of efforts to promote family planning and preconception care.

In contrast to our results, a small study exploring why women did not respond to an invitation to attend for preconception care found that despite generally subscribing to the value of preventive behaviours and a healthy lifestyle, women perceived themselves as having sufficient knowledge of preconception care issues and/or not being at risk. Some also misunderstood the aim of preconception care as being advice about infertility [17]. This perception of sufficient knowledge was only consistent for women in our study who had previously had children. For these reasons, asking women to complete existing screening tools for preconception risk factors may assist in overcoming these barriers and facilitate the provision of preconception care delivery and uptake [18].

An important finding in our study is the way women perceived the role of the GP with regards to prevention. Lower SES and rural women were reluctant to use GPs time for preventive activity that they perceived as only involving counselling, feeling that they would be depriving those in need of more urgent medical care. Higher SES women on the other hand felt that preconception care was not in the remit of practice or skills of a GP and turned to alternative health professionals such as naturopaths. We suggest that these findings might be relevant to the implementation of other forms of prevention, and that patient perceptions of the role of GPs in prevention requires further exploration.

Bille and Anderson (2009) suggest that preconception counselling should be part of a scheduled preventive health program, acknowledging that couples would need to seek out and attend such programs [5]. In contrast, our study suggests women need to be receptive to preconception care in order to access it and that they would like to have it promoted to them by their GP and by other means such as letters.

Whilst this study highlights the perspectives of women of reproductive age, several limitations exist. Firstly, findings of this study are limited in generalisability, given the small sample size and the specific geographic location of focus groups. Women who participated in this study may not be representative of the population sampled and different perspectives may exist for groups of women not specifically targeted in this study i.e. teenagers, single women, middle income women, and women with co-morbidities or substance addiction. Furthermore, perspectives of women also may be different in areas where active policy and public health campaigns exist that specifically address preconception care and periconceptional folate supplementation. Secondly, as periconceptional folate supplementation is one aspect of preconception care, the findings in relation to it are somewhat lessened as women didn't fully appreciate preconception care in general.

## Conclusion

Having identified the views of women and the fact that multiple barriers and enablers to the uptake of preconception care and periconceptional folate supplementation exist, we believe that further research is needed to identify which of these are the most important and amenable to change. Prior to developing an intervention to improve preconception care, it will also be important to; determine the views of GPs and other health professionals about the barriers they experience to preconception care delivery and how these relate to what women describe, and to understand the theoretical basis involved in changing health professionals behaviour. For the delivery and uptake of preconception care to improve, it essential that there is a demand from both parties. This more rigorous approach to designing interventions which better target the barriers to practice change may result in more success in implementing preconception care [19-21].

## Competing interests

The authors declare that they have no competing interests.

## Authors' contributions

DM developed the concept and design of the study, and was responsible for data acquisition. DM and AC analysed the data, interpreted the results, drafted the manuscript, and approved the final manuscript. DM is the guarantor.

## Pre-publication history

The pre-publication history for this paper can be accessed here:

http://www.biomedcentral.com/1471-2458/10/786/prepub
